# Prospective Observational Study of Implantable Cardioverter‐Defibrillators in Primary Prevention of Sudden Cardiac Death: Study Design and Cohort Description

**DOI:** 10.1161/JAHA.112.000083

**Published:** 2013-02-22

**Authors:** Alan Cheng, Darshan Dalal, Barbara Butcher, Sanaz Norgard, Yiyi Zhang, Timm Dickfeld, Zayd A. Eldadah, Kenneth A. Ellenbogen, Eliseo Guallar, Gordon F. Tomaselli

**Affiliations:** 1 Department of Medicine, Johns Hopkins University, Baltimore, MD (A.C., D.D., B.B., S.N., G.F.T.); 2 Department of Epidemiology, Johns Hopkins University, Baltimore, MD (D.D., Y.Z., E.G.); 3 Department of Medicine, University of Maryland, Baltimore, MD (T.D.); 4 Washington Hospital Center, Washington, DC (Z.A.E.); 5 Virginia Commonwealth University Pauley Heart Center, Richmond, VA (K.A.E.); 6 National Center for Cardiovascular Research, Madrid, Spain (E.G.)

**Keywords:** cardiomyopathy, implantable cardioverter‐defibrillator, sudden death, ventricular tachyarrhythmias

## Abstract

**Background:**

Primary‐prevention implantable cardioverter‐defibrillators (ICDs) reduce total mortality in patients with severe left ventricular systolic function. However, only a minority of patients benefit from these devices. We designed the Prospective Observational Study of Implantable Cardioverter‐Defibrillators (PROSE‐ICD) to identify risk factors and enhance our understanding of the biological mechanisms that predispose to arrhythmic death in patients undergoing ICD implantation for primary prevention of sudden death.

**Methods and Results:**

This is a multicenter prospective cohort study with a target enrollment of 1200 patients. The primary end point is ICD shocks for adjudicated ventricular tachyarrhythmias. The secondary end point is total mortality. All patients undergo a comprehensive evaluation including history and physical examination, signal‐averaged electrocardiograms, and blood sampling for genomic, proteomic, and metabolomic analyses. Patients are evaluated every 6 months and after every known ICD shock for additional electrocardiographic and blood sampling. As of December 2011, a total of 1177 patients have been enrolled with more nonwhite and female patients compared to previous randomized trials. A total of 143 patients have reached the primary end point, whereas a total of 260 patients died over an average follow‐up of 59 months. The PROSE‐ICD study represents a real‐world cohort of individuals with systolic heart failure receiving primary‐prevention ICDs.

**Conclusions:**

Extensive electrophysiological and structural phenotyping as well as the availability of serial DNA and serum samples will be important resources for evaluating novel metrics for risk stratification and identifying patients at risk for arrhythmic sudden death.

**Clinical Trial Registration:**

URL: http://clinicaltrials.gov/ Unique Identifier: NCT00733590.

## Introduction

Sudden cardiac death (SCD) is the most common cause of death in the United States, affecting >300 000 individuals yearly.^1–3^ SCD is most often the result of ventricular tachyarrhythmias that occur secondary to a complex interplay between a susceptible myocardial substrate typically affected by ischemic or nonischemic cardiomyopathy and a transient trigger.^4^ Primary prevention implantable cardioverter‐defibrillators (ICDs) have been shown to reduce total mortality in high‐risk patients.^5–6^ Early efforts aimed at identifying individuals at greatest risk for SCD included the use of several invasive and noninvasive metrics including electrophysiology studies, signal‐averaged electrocardiograms, heart rate variability, heart rate turbulence, measurement of microvolt T‐wave alternans, and measurements of repolarization lability.^7–11^ Although some showed early promise, the most robust metric has been reductions in the left ventricular ejection fraction. Unfortunately, this metric is neither highly sensitive nor specific and does not take into consideration other genetic or electrophysiological determinants for arrhythmogenesis. As a result, there is substantial interest in identifying more reliable predictors that could help to discriminate which patients are most likely to benefit.

The Prospective Observational Study of Implantable Cardioverter‐Defibrillators (PROSE‐ICD) is a multicenter prospective observational study of patients undergoing ICD or cardiac resynchronization therapy (CRT) implantation for primary prevention of SCD. Its aim is to identify risk factors and enhance our understanding of the biological mechanisms that predispose to arrhythmic SCD, with the hope of developing a paradigm to identify individuals who stand to benefit the most and the least from primary prevention ICDs. Patients undergo extensive phenotyping and biobanking of DNA and serum at the time of implantation, at 6‐month intervals throughout follow‐up, and after ICD shock events. This article presents an overview of the PROSE‐ICD design, a descriptive analysis of the demographics of the study participants, and anticipated event rates and enrollment plans.

## Methods

### Study Population

PROSE‐ICD is a multicenter prospective cohort study (clinicaltrials.gov identifier NCT00733590) of patients undergoing ICD implantation for primary prevention of SCD on the basis of criteria outlined in current practice guidelines.^12^ The study is being conducted at 4 centers in the United States: the Johns Hopkins Hospital and Bayview Medical Center, Baltimore, MD; the University of Maryland Hospital, Baltimore, MD; the Washington Hospital Center, Washington, DC; and the Virginia Commonwealth University Hospital, Richmond, VA.

The study population includes primary prevention ICD or CRT recipients between 18 and 80 years of age fulfilling 1 of 2 criteria: (1) ischemic cardiomyopathy (myocardial infarction occurring >40 days prior to implant and no revascularization within the past 90 days) with an ejection fraction of ≤30% and (2) ischemic or nonischemic cardiomyopathy with an ejection fraction ≤35% and stable heart failure symptoms (New York Heart Association [NYHA] Class II to III) on optimal pharmacotherapy (Table 1). In addition, CRT patients were included if they demonstrated an ejection fraction of ≤35%, NYHA Class III to IV heart failure symptoms, and a QRS width of >120 ms. No requirement is specified regarding QRS morphology. Patients are excluded if the ICD or CRT was implanted for secondary prevention, if they had a permanent pacemaker or a preexisting class 1 indication for pacemaker implantation, NYHA Class IV heart failure (unless they were undergoing implantation of a CRT device), or fulfillment of any Class III indication for primary prevention ICD implantation as outlined in current practice guidelines.^12^ Patients are enrolled prior to ICD implantation but considered part of the study cohort only after successful ICD placement. PROSE‐ICD recruitment started in December 2003 and is ongoing with a target enrollment of 1200. This study has been approved by the institutional review boards of all participating hospitals. All participants have provided written informed consent.

**Table 1.** Criteria for Inclusion in the PROSE‐ICD Cohort

Inclusion Criteria	Exclusion Criteria
18 to 80 years of age	ICD implantation for secondary prevention
History of acute myocardial infarction ≥40 days old (confirmed by persistent pathologic Q waves on ECG, clinical reports of CPK‐MB >3 times the upper limit of normal, or a fixed perfusion defect on nuclear imaging) with an ejection fraction (EF) ≤30% and no history of revascularization within the last 3 months.	Patients with a permanent pacemaker or a preexisting class 1 indication for pacemaker implantation
	Patients with New York Heart Association Class IV heart failure (unless undergoing CRT implantation)
History of ischemic or nonischemic left ventricular systolic dysfunction with stable NYHA Class II to III heart failure symptoms for ≥3 months on optimal pharmacotherapy and an EF ≤35%. For CRT patients, EF ≤35%, QRS >120 ms, NYHA Class III to IV heart failure symptoms on optimal pharmacotherapy	Patients with history of a confirmed myocardial within 40 days of implant or revascularization within the last 3 months
	Patients fulfilling class III indications for primary prevention ICD implantation
	Unsuccessful ICD implantation
	Patient unable or unwilling to provide informed consent

PROSE‐ICD indicates Prospective Observational Study of Implantable Cardioverter‐Defibrillators; ECG, electrocardiogram; CRT, cardiac resynchronization therapy; NYHA, New York Heart Association; CPK‐MB, creatine kinase MB band.

### Preimplantation Assessment and Serum Processing

At enrollment and prior to ICD implantation, each patient undergoes a comprehensive history and cardiovascular physical examination, electrocardiographic (ECG) evaluation, cardiac imaging to assess ejection fraction, and a blood draw. ECGs are recorded digitally (Norav Medical, Ontario, Canada) and include a standard 12‐lead recording as well as a high‐resolution 5‐minute rhythm strip using 3 orthogonal leads. Adjudicated automated readings of the 12‐lead ECGs are performed for rhythm determination and conduction abnormalities. The rhythm strip is analyzed by computer‐based algorithms for signal averaging and for measuring heart rate and QT variability parameters. Heart rate and QT variability analyses are performed in the time and frequency domains after excluding ECGs exhibiting frequent atrial or ventricular ectopy, atrial fibrillation, or ventricular pacing. In addition, patients with bundle branch block are excluded from QT variability analyses.

The blood draw and processing are standardized. Peripheral blood is collected and allowed to stand at room temperature for 1 hour to minimize the variance in the time from collection to processing. Blood in nonanticoagulated (red‐top) tubes is centrifuged at 1500 rpm for 5 minutes. The serum is then transferred into tubes containing 200‐ to 500‐μL aliquots, frozen in liquid nitrogen and stored at −80°C. Buffy coats are also collected and after a similar low‐speed spin, are transferred into 100‐μL aliquots prior to −80°C storage. Routine clinical laboratory tests are performed and the specific tests determined by the treating physicians. Additional blood is collected for future analysis as part of this study. There are no prespecified blood tests performed on the study samples, but biomarker, metabolomic, and genomic analyses are planned. The specific platforms for genetic testing are not prespecified but will be focused on analyzing large numbers of single‐nucleotide polymorphisms among other things.

### ICD Implantation and Programmed Stimulation

Device implantation is performed only in patients without clinical evidence of active myocardial ischemia, decompensated heart failure, or infection. All procedures are performed in the standard clinical fashion either with conscious sedation or general anesthesia. In cases of CRT devices, left ventricular pacing leads are targeted to a lateral or posterolateral branch of the great cardiac vein. The final location of the left ventricular pacing lead is assessed using saved fluoroscopic images at the time of device implantation. Toward the end of the procedure, physicians are encouraged to perform programmed stimulation through the device. Programmed ventricular stimulation is performed at 2 drive trains (350 and 500 ms) with up to 3 extrastimuli. The minimum extrastimulus coupling interval is 200 ms. When the ventricular effective refractory period (VERP) is reached for a given extrastimulus, the coupling interval of the preceding extrastimulus is decremented by 10‐ms intervals until VERP is reached or a coupling interval of 200 ms. This process is repeated for all extrastimuli. Morphology and cycle lengths of all sustained ventricular arrhythmias induced are recorded. Data on ICD generator and leads implanted, tachycardia therapy cutoff rates and therapies programmed, use of supraventricular arrhythmia discriminators, and sensitivity settings are also noted.

### Patient Follow‐Up Visits, Adjudication of ICD Arrhythmias, and Censoring Events

Patients are evaluated every 6 months after implantation and soon after any ICD shock event reported by the patient (Figure). At each visit, study participants undergo a focused cardiac history and physical exam, digital ECG recording, and blood draws as described above. The ICD is also interrogated for device performance metrics and recorded arrhythmic events. All stored electrograms from delivered ICD therapies are collected and adjudicated by 2 clinical cardiac electrophysiologists blinded to patient identifiers or other clinical information. For every recorded event, each electrophysiologist independently determines the rhythm at the time of initial detection and after therapy delivery. If there is disagreement with the diagnosed rhythm at the time of initial detection, the episode is then reviewed by a third electrophysiologist for final adjudication. Patients who miss their scheduled visits or are unable to return to the enrolling center for follow‐up are contacted by phone. During these phone interviews, current health status and medication use are updated as well as any interim cardiovascular‐related events. Every effort is made to obtain source documentation of pertinent hospitalizations.

**Figure 1.**
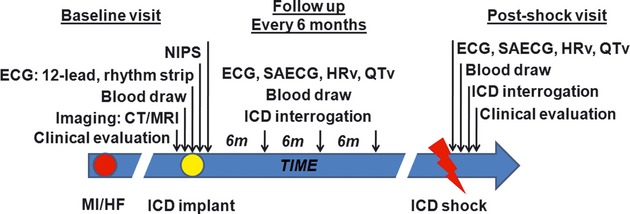
Study design of PROSE‐ICD. Baseline measurements include comprehensive history, digitized signal‐averaged ECG, 12‐lead baseline ECG, blood collection, and in some patients, cardiac computed tomography (CT) with contrast or cardiac magnetic resonance imaging (MRI) with gadolinium‐based delayed hyperenhancement. Patients are routinely evaluated either in person or via phone call every 6 months. Soon after an ICD shock, patients are encouraged to return to the clinic for further evaluation. At the visit after the shock, the device is interrogated and the event downloaded for adjudication. A 12‐lead ECG, signal‐averaged ECG, and blood collection are also collected again. PROSE‐ICD indicates Prospective Observational Study of Implantable Cardioverter‐Defibrillators; ECG, electrocardiogram; NIPS, noninvasive programmed stimulation through the ICD; MI/HF, index myocardial infarction or initial diagnosis of heart failure; SAECG, signal‐averaged ECG; HRv, heart rate variability analysis; QTv, QT interval variability analysis.

### Primary and Secondary End Points

The primary outcome of PROSE‐ICD is ICD shocks for adjudicated ventricular tachyarrhythmias. The secondary outcome is all‐cause mortality. It is expected that some patients will have multiple ICD shocks for ventricular tachyarrhythmias. For the purposes of the analysis, the first confirmed instance will be used *unless* it was preceded by an ICD shock for supraventricular tachyarrhythmias within a 24‐hour period. We are qualifying this definition because we cannot be absolutely sure that there was no residual effect of the first “inappropriate” shock on the development of the subsequent “appropriate” shock. Deaths are ascertained by phone contact with the next of kin and by searches of the National Death Index. Next of kin interviews regarding the circumstances surrounding the death, death certificates, and medical documentation around the time of death including ICD interrogations whenever possible are collected. This information is subsequently reviewed and adjudicated by 2 independent cardiologists. Each death event is adjudicated on the basis of modified Hinkle–Thaler criteria.^13^

### Statistical Analysis

Patients are considered part of the cohort after successful ICD implant. For time‐to‐event analysis for the primary end point, patients are censored at the time of death, ICD explantation, left ventricular assist device implantation or heart transplantation, or at the last date of contact among those still alive. For time‐to‐event analysis for all‐cause mortality (secondary end point), follow‐up is censored at the time of ICD explantation, left ventricular assist device implantation or heart transplantation, or at the last date of contact or the date of death in the National Death Index. These events are selected on the basis of their likelihood of altering the patient's underlying cardiac disease process and hence their risk for ICD shocks. The Kaplan–Meier method will be used to estimate the cumulative incidence of these events. Incidence rates for a given event will be calculated by taking the ratio of the total number of events and the total number of person‐years of follow‐up contributed by cohort participants. Hazard ratios of study outcomes adjusted for other participant characteristics will be estimated using proportional hazards regression models.

### Sample Size and Power Estimates

On the basis of prior randomized clinical trials,^5–6^ the annualized primary end point event rate is estimated at 6%. Annual loss from censoring and losses to follow‐up are estimated at 5%. We calculate that a target enrollment of 1200 patients will result in ≈362 primary end point events by the end of follow‐up and will allow us to detect an estimated hazard ratio of ≥1.85 when comparing extreme quartiles of an independent predictor of ICD shocks with 80% power and a probability of type I error of 5% (2‐sided).

## Results

### Study Population

As of December 31, 2011, PROSE‐ICD has enrolled 1177 patients. Although their characteristics are similar to the patients enrolled in MADIT II and SCD‐HeFT, there are some notable differences (Table 2). Overall, individuals are predominantly male with a relatively balanced distribution of cardiomyopathy etiology. The mean±SD ejection fraction is 23±8%, with a majority of individuals exhibiting New York Heart Association Class I to II heart failure symptoms. The proportion of nonwhite patients is 43%. The baseline prevalence of hypertension and diabetes is 63% and 35%, respectively. Overall, there are high rates of heart failure medication usage at the time of enrollment with 90% of individuals taking β‐blockers and 88% taking angiotensin‐converting enzyme inhibitors (ACE‐Is) or angiotensin receptor blockers. Enrollment at the 4 sites reveals differences in demographics across the sites (Table 3). The Johns Hopkins University site shows the highest proportion of white participants and a lower prevalence of diabetes and hypertension. There is no difference in either the percentage of patients with ischemic cardiomyopathy or significant differences in left ventricular ejection fraction or NYHA class function across the sites. ICD programming is left to the discretion of the implanting physician. To date, the average cutoff zone is 184.8 beats per minute (bpm) with a standard deviation of 15.4 bpm.

**Table 2.** Qualitative Comparison of Demographics and Clinical Characteristics of PROSE‐ICD (as of December 31, 2011) With ICD Arms of MADIT‐II and SCD‐HeFT Trial. Total Number of Patients Enrolled in MADIT II and SCD‐HeFT Are 1232 and 2521, Respectively. Description Below Only Includes Those in ICD Arms of the Studies

Characteristic	PROSE‐ICD (n=1177)	MADIT‐II^6^ (n=742)	SCD‐HeFT^5^ (n=829)
Age, y	61±13	64±10	60.1*
Sex, male	73	84	77
Race, nonwhite	43	0	23
Smoking	67	80	—
Diabetes	35	33	31
Hypertension	63	53	55
LVEF	23±8	23±5	24*
NYHA class			
Class I	17	35	0
Class II	43	35	70
Class III	38	25	30
Class IV	1	5	0
Cardiomyopathy, ischemic	54	100	52

Data expressed as mean±SD or percentage, unless otherwise indicated. PROSE‐ICD indicates Prospective Observational Study of Implantable Cardioverter‐Defibrillators; MADIT‐II, Multicenter Automatic Defibrillator Implantation Trial‐II; SCD‐HeFT, Sudden Cardiac Death in Heart Failure Trial; LVEF, left ventricular ejection fraction; NYHA, New York Heart Association.

* Median.

**Table 3.** Demographics and Clinical Characteristics of PROSE‐ICD by Enrolling Center as of December 31, 2011

Characteristic	JHU (n=730)	UMD (n=86)	VCU (n=72)	WHC (n=289)	Total (n=1177)
Age, y	60±13	62±14	58±13	62±12	61±13
Sex, male	72	82	64	75	73
Race, white	67	40	39	40	57
Smoking	69	65	44	68	67
Diabetes	30	44	43	42	35
Hypertension	51	81	76	83	63
LVEF, %	22±8	23±9	23±6	24±7	23±8
NYHA class					
Class I	17	7	14	23	17
Class II	36	45	49	60	43
Class III	46	48	36	17	38
Class IV	1	0	0	0	1
Cardiomyopathy, ischemic	52	55	51	59	54
Medication use					
Beta‐blocker	88	87	86	92	90
ACE inhibitor/ARB	88	88	85	87	88
Statin	66	65	65	79	70
Antiarrhythmics	9	6	3	12	10
Aldosterone antagonist	25	27	29	23	25

Values expressed as mean±SD or frequency (%). PROSE‐ICD indicates Prospective Observational Study of Implantable Cardioverter‐Defibrillators; JHU, Johns Hopkins University; UMD, University of Maryland; VCU, Virginia Commonwealth University, WHC, Washington Hospital Center; LVEF, left ventricular ejection fraction; NYHA, New York Heart Association; ACE, angiotensin‐converting enzyme; ARB, angiotensin receptor blocker.

### Current Study End Points

As of December 31, 2011, a total of 234 patients have experienced an ICD shock over an average of 59±23 months of follow‐up. Among them, 143 experienced ICD shocks for ventricular tachyarrhythmias.

## Discussion

Sudden cardiac death remains the most common mode of death in the United States and other developed countries. The use of primary prevention ICDs has been shown to reduce total mortality in individuals with severe dilated cardiomyopathy, but only a minority of patients receive appropriate ICD therapies. Furthermore, current rates of periprocedural complications,^14^ device malfunctions^15^ and the incidence of inappropriate ICD shocks^16–17^ have led some to question whether the benefits outweigh the risks.^18–19^ Hence, it is ever more important to accurately identify individuals who stand to benefit the most from this intervention.

The optimal strategy for risk stratification of primary prevention individuals at greatest risk for sudden death is still poorly defined. Current strategies rely in large measure on the left ventricular ejection fraction, but limited sensitivity and specificity have led to the proposal of other noninvasive measurements^20^ such as microvolt T‐wave alternans,^21^ magnetic resonance imaging,^22^ serum‐based biomarkers,^23^ or a combination of these metrics.^24^ Additional validation in large cohorts will be required prior to widespread adoption of these techniques in clinical practice.^25^

PROSE‐ICD was conceived with the overall aim of better understanding the biological pathways predisposing to SCD. We will use extensive phenotyping of study participants including genetic, proteomic, metabolomic, electrophysiological, and epidemiological markers to help establish a risk stratification schema that will identify patients at highest and lowest risk for SCD. This study is one of the largest, most extensively phenotyped cohorts of patients undergoing prophylactic ICD implantation. The stringent enrollment criteria and phenotyping accompanied by routine data‐quality checks will provide good internal and external validity of the data.

Although the current demographic makeup of this study is similar to previous landmark clinical trials,^5–6^ there are some differences. First, the percentages of women and nonwhites are 27% and 43%, respectively. This is in contrast to patients from the SCD‐HeFT study, whose enrollment was 23% women and 23% nonwhite subjects. PROSE‐ICD will thus provide insights into the effects of sex and race that cannot be obtained from other studies because of enrollment limitations. Second, the use of heart failure medications at the time of enrollment is optimal, with ≈90% of individuals on β‐blockers and 88% on ACE‐Is. Whether outcomes in patients with ICDs are modulated by optimal heart failure medications remains to be seen. Third, this study reflects current clinical practice patterns and did not require implanting physicians to adopt a prespecified device programming strategy (eg, utilization of antitachycardia pacing, programming several tachyarrhythmic therapy zones). Hence, the findings from this study stand to better reflect how patients in the general population do over time and provide us with real‐world outcomes. Lastly, the plans for extensive clinical phenotyping and blood collection will allow us to prospectively follow patients and potentially identify novel markers of risk for arrhythmic events.

### Potential Limitations

This study is a prospective, observational study, and inherent limitations of this design as well as variables not currently measured at the time of enrollment may have an effect on the interpretability of the findings. In addition, we have focused on enrollment of individuals with evidence of systolic heart failure. Hence, findings from this study may not apply to the smaller group of individuals with preserved systolic function undergoing primary prevention ICD implantation such as long QT syndrome, early‐stage cardiac sarcoidosis, and other inherited genetic channelopathies. Despite these limitations, we believe that the findings from this study will have significant implications on SCD risk assessment and management as this cohort represents the largest, most extensively phenotyped group of individuals undergoing ICD implantation for primary prevention of sudden death.

## Conclusions

The PROSE‐ICD study represents a typical cohort of patients receiving ICDs for primary prevention of SCD on the basis of current practice guidelines. Although the clinical demographics appear to be similar to previously published randomized trials, there are some notable differences. PROSE‐ICD will allow us to identify subgroups that stand to benefit the most from these devices and perhaps to craft novel strategies for risk stratification of patients potentially at risk for sudden death.

## References

[1] Zheng ZJ Croft JB Giles WH Mensah GA Sudden cardiac death in the United States, 1989–1998. Circulation. 2001; 104:2158-2163 11684624 10.1161/hc4301.098254

[2] Roger VL Go AS Lloyd‐Jones DM Benjamin EJ Berry JD Borden WB Bravata DM Dai S Ford ES Fox CS Fullerton HJ Gillespie C Hailpern SM Heit JA Howard VJ Kissela BM Kittner SJ Lackland DT Lichtman JH Lisabeth LD Makuc DM Marcus GM Marelli A Matchar DB Moy CS Mozaffarian D Mussolino ME Nicol G Paynter NP Soliman EZ Sorlie PD Sotoodehnia N Turan TN Virani SS Wong ND Woo D Turner MB American Heart Association Statistics Committee Stroke Statistics Subcommittee Heart disease and stroke statistics—2012 update: a report from the American Heart Association. Circulation. 2012; 125:e2-e220 22179539 10.1161/CIR.0b013e31823ac046 PMC4440543

[3] Chugh SS Jui J Gunson K Stecker EC John BT Thompson B Ilias N Vickers C Dogra V Daya M Kron J Zheng ZJ Mensah G McAnulty J Current burden of sudden cardiac death: multiple source surveillance versus retrospective death certificate‐based review in a large US community. J Am Coll Cardiol. 2004; 44:1268-1275 15364331 10.1016/j.jacc.2004.06.029

[4] Zipes DP Wellens HJ Sudden cardiac death. Circulation. 1998; 98:2334-2351 9826323 10.1161/01.cir.98.21.2334

[5] Bardy GH Lee KL Mark DB Poole JE Packer DL Boineau R Domanski M Troutman C Anderson J Johnson G McNulty SE Clapp‐Channing N Davidson‐Ray LD Fraulo ES Fishbein DP Luceri RM Ip JH Sudden Cardiac Death in Heart Failure Gtrial (SCD‐HeFT) Investigators Amiodarone or an implantable cardioverter‐defibrillator for congestive heart failure. N Engl J Med. 2005; 352:225-237 15659722 10.1056/NEJMoa043399

[6] Moss AJ Zareba W Hall WJ Klein H Wilber DJ Cannom DS Daubert JP Higgins SL Brown MW Andrews ML Multicenter Automatic Defibrillator Implantation Trial II Investigators Prophylactic implantation of a defibrillator in patients with myocardial infarction and reduced ejection fraction. N Engl J Med. 2002; 346:877-883 11907286 10.1056/NEJMoa013474

[7] Thomas KE Josephson ME The role of electrophysiology study in risk stratification of sudden cardiac death. Prog Cardiovasc Dis. 2008; 51:97-105 18774009 10.1016/j.pcad.2008.05.001

[8] Stein KM Noninvasive risk stratification for sudden death: signal‐averaged electrocardiography, nonsustained ventricular tachycardia, heart rate variability, baroreceptor sensitivty, and QRS duration. Prog Cardiovasc Dis. 2008; 51:106-117 18774010 10.1016/j.pcad.2007.10.001

[9] La Rovere MT Pinna GD Hohnloser SH Marcus FI Mortara A Nohara R Bigger JT Camm AJ Schwartz PJ ATRAMI Investigators (Autonomic Tone and Reflexes After Myocardial Infarction) Baroreflex sensitivity and heart rate variability in identification of patients at risk for life‐threatening arrhythmias: implications for clinical trials. Circulation. 2001; 103:2072-2077 11319197 10.1161/01.cir.103.16.2072

[10] Costantini O Hohnloser SH Kirk MM Lerman BB Baker JH Sethuraman B Dettmer MM Rosenbaum DS ABCD Trial Investigators The ABCD (Alternans Before Cardioverter‐Defibrillator) Trial: strategies using T‐wave alternans to improve efficiency of sudden cardiac death prevention. J Am Coll Cardiol. 2009; 53:471-479 19195603 10.1016/j.jacc.2008.08.077

[11] Chow T Kereiakes DJ Onufer J Woelfel A Gursoy S Peterson BJ Brown ML Pu W Benditt DG MASTER Trial Investigators Does microvolt T‐wave alternans testing predict ventricular tachyarrhythmias in patients with ischemic cardiomyopathy and prophylactic defibrillators?: the MASTER (Microvolt T Wave Alternans Testing for Risk Stratification of Post‐Myocardial Infarction Patients) trial. J Am Coll Cardiol. 2008; 52:1607-1615 18992649 10.1016/j.jacc.2008.08.018

[12] Epstein AE DiMarco JP Ellenbogen KA Estes NA III Freedman RA Gettes LS Gillinov AM Gregoratos G Hammill SC Hayes DL Hlatky MA Newby LK Page RL Shoenfeld MH Silka MJ Stevenson LW Sweeney MO Smith SC Jr Jacobs AK Adams CD Anderson JL Buller CE Creager MA Ettinger SM Faxon DP Halperin JL Hiratzka LF Hunt SA Krumholz HM Kushner FG Lytle BW NIshimura RA Ornato JP Page RL Riegel B Tarkington LG Yancy CW ACC/AHA/HRS 2008 Guidelines for Device‐Based Therapy for Cardiac Rhythm Abnormalities: a report of the American College of Cardiology/American Heart Association Task Force on Practice Guidelines. Circulation. 2008; 2008:e350-e408 23255456 10.1161/CIR.0b013e318276ce9b

[13] Hinkle LE Jr Thaler HT Clinical classification of cardiac deaths. Circulation. 1982; 65:457-464 7055867 10.1161/01.cir.65.3.457

[14] Haines DE Wang Y Curtis J Implantable cardioverter‐defibrillator registry risk score models for acute procedural complications or death after implantable cardioverter‐defibrillator implantation. Circulation. 2011; 123:2069-2076 21537001 10.1161/CIRCULATIONAHA.110.959676

[15] Maisel WH Pacemaker and ICD generator reliability: meta‐analysis of device registries. JAMA. 2006; 295:1929-1934 16639052 10.1001/jama.295.16.1929

[16] Poole JE Johnson GW Hellkamp AS Anderson J Callans DJ Raitt MH Reddy RK Marchlinski FE Yee R Guarnieri T Talajic M Wilber DJ Fishbein DP Packer DL Mark DB Lee KL Bardy GH Prognostic importance of defibrillator shocks in patients with heart failure. N Engl J Med. 2008; 359:1009-1017 18768944 10.1056/NEJMoa071098 PMC2922510

[17] van Rees JB Borleffs CJ de Bie MK Stijnen T van Erven L Bax JJ Schalij MJ Inappropriate implantable cardioverter‐defibrillator shocks: incidence, predictors, and impact on mortality. J Am Coll Cardiol. 2011; 57:556-562 21272746 10.1016/j.jacc.2010.06.059

[18] Tung R Zimetbaum P Josephson ME A critical appraisal of implantable cardioverter‐defibrillator therapy for prevention of sudden death. J Am Coll Cardiol. 2008; 52:1111-1121 18804736 10.1016/j.jacc.2008.05.058

[19] Kramer DB Buxton AE Zimetbaum PJ Time for a change—a new approach to ICD replacement. N Engl J Med. 2012; 366:291-293 22276818 10.1056/NEJMp1111467

[20] Chugh SS Early identification of risk factors for sudden cardiac death. Nat Rev Cardiol. 2010; 7:318-326 20421887 10.1038/nrcardio.2010.52 PMC3052394

[21] Chow T Kereiakes DJ Bartone C Booth T Schloss EJ Waller T Chung E Menon S Nallamothu BK Chan PS Microvolt T wave alternans identifies patients with ischemic cardiomyopathy who benefit from implantable cardioverter‐defibrillator therapy. J Am Coll Cardiol. 2007; 49:50-58 17207722 10.1016/j.jacc.2006.06.079

[22] Kwon DH Halley CM Carrigan TP Zysek V Popovic ZB Setser R Schoenhagen P Starling RC Flamm SD Desai MY Extent of left ventricular scar predicts outcomes in ischemic cardiomyopathy patients with significantly reduced systolic function: a delayed hyperenhancement cardiac magnetic resonance study. JACC Cardiovasc Imaging. 2009; 2:34-44 19356530 10.1016/j.jcmg.2008.09.010

[23] Havmöller R Chugh SS Plasma biomarkers for prediction of sudden cardiac death: another piece of the risk stratification puzzle? Circ Arrhythm Electrophysiol. 2012; 5:237-243 22334431 10.1161/CIRCEP.111.968057 PMC3622872

[24] Wu KC Gerstenblith G Guallar E Marine JE Dalal D Cheng A Marban E LIma JA Tomaselli GF Weiss RG Combined cardiac magnetic resonance imaging and C‐reactive protein levels identify a cohort at low risk for defibrillator firings and death. Circ Cardiovasc Imaging. 2012; 5:178-186 22267750 10.1161/CIRCIMAGING.111.968024 PMC3330427

[25] Dickfeld T Pursuing the “holy grail”. Circ Cardiovasc Imaging. 2012; 5:167-170 22438421 10.1161/CIRCIMAGING.112.972935

